# An Open-Access Modeled Passenger Flow Matrix for the Global Air Network in 2010

**DOI:** 10.1371/journal.pone.0064317

**Published:** 2013-05-15

**Authors:** Zhuojie Huang, Xiao Wu, Andres J. Garcia, Timothy J. Fik, Andrew J. Tatem

**Affiliations:** 1 Department of Geography, University of Florida, Gainesville, Florida, United States of America; 2 Emerging Pathogens Institute, University of Florida, Gainesville, Florida, United States of America; 3 Department of Statistics, University of Florida, Gainesville, Florida, United States of America; 4 Fogarty International Center, National Institutes of Health, Bethesda, Maryland, United States of America; 5 Department of Geography and Environment, University of Southampton, Highfield, Southampton, United Kingdom; University of Warwick, United Kingdom

## Abstract

The expanding global air network provides rapid and wide-reaching connections accelerating both domestic and international travel. To understand human movement patterns on the network and their socioeconomic, environmental and epidemiological implications, information on passenger flow is required. However, comprehensive data on global passenger flow remain difficult and expensive to obtain, prompting researchers to rely on scheduled flight seat capacity data or simple models of flow. This study describes the construction of an open-access modeled passenger flow matrix for all airports with a host city-population of more than 100,000 and within two transfers of air travel from various publicly available air travel datasets. Data on network characteristics, city population, and local area GDP amongst others are utilized as covariates in a spatial interaction framework to predict the air transportation flows between airports. Training datasets based on information from various transportation organizations in the United States, Canada and the European Union were assembled. A log-linear model controlling the random effects on origin, destination and the airport hierarchy was then built to predict passenger flows on the network, and compared to the results produced using previously published models. Validation analyses showed that the model presented here produced improved predictive power and accuracy compared to previously published models, yielding the highest successful prediction rate at the global scale. Based on this model, passenger flows between 1,491 airports on 644,406 unique routes were estimated in the prediction dataset. The airport node characteristics and estimated passenger flows are freely available as part of the Vector-Borne Disease Airline Importation Risk (VBD-Air) project at: www.vbd-air.com/data.

## Introduction

Demand for travel has boosted the growth of the global air travel network at an unprecedented rate. In the past 20–30 years, the network has expanded dramatically with a steady growth rate of 4–5% per year [1], accompanied by a nearly 9% annual growth rate of passenger and freight traffic [2]. In 2011, the worldwide international and domestic passenger kilometers transported reached a record-high of 5.2 trillion kilometers [3]. The large volumes of air traffic, result in profound impacts on commodity trade [4], regional development [5], cultural communication [6], disease importation [7], [8] and species invasion [9]–[11]. As humans and commodities are transported at exceptional rates through aviation compared to other modes of transportation, how these patterns impact the socioeconomic, environmental and epidemiological landscape is of significant interest [7], [9], [11], [12].

Quantifying the volume of passengers on the air travel network is critical to understanding the complicated spatial interaction between origin and the destination cities [7], [8]. Previously, studies from a range of fields [9]–[11], [13]–[16] have made use of data from the International Air Transport Association (IATA) or the International Civil Aviation Organization (ICAO). These data are often restricted to scheduled flight plus seat capacity information on routes. However, not all commercial flights operate at full capacity; and such data often overestimate the passenger numbers on affected routes [7]. Moreover, capacity data provide information on only point-to-point connection; thus, travel patterns that require a stopover and transfer of planes are not captured [17]. Although origin-destination data derived from air ticket sales are available (e.g. http://www.iata.org/ps/intelligence_statistics/paxis/pages/index.aspx ), such data are expensive for research purposes, running to many tens of thousands of dollars, and can require significant legal and confidentiality agreements for data usage. Other databases of international flow by pair-wise airports are held by private companies (e.g. Marketing Information Data Transfer, http://ma.aspirion.aero/midt). These proprietary data bases are costly and difficult to obtain; with payment required repeatedly to maintain the latest data. Here we aim to outline a modeling framework to produce open-access estimates of global air traffic flows for research purposes that can be regularly updated.

Spatial interaction models have been utilized to estimate the volume of passengers given an origin and destination city where data are lacking [4], [14], [15], [18]–[25]. The most common of which is the gravity-type model, which incorporates drivers such as the site characteristics of origins and destinations, and measures of “locational separation” to depict the interaction between origins and destinations for purposes of estimating flows. As Grosche et al [25] summarized, commonly used drivers in the spatial interaction model to estimate the air traffic include 1) socio-economic characteristics of origins and destinations, such as population, income, GDP, urban infrastructure, education level, and 2) service-related factors such as the quality (e.g., flight frequency, plane size and air fares) and the market demand of airline service. The locational separation is usually calibrated by the distance or travel time separating origins and destinations. The gravity model provides a solid theoretical and practical background on understanding the movement of populations since it explicitly captures the absolute and relative spatial relationship of origins and destinations [19].

The utilization of network characteristics sheds light on the identification of air service factors in the gravity model for flow estimation, since 1) the layout of the global air travel network follows the “hub-and-spoke” network model and 2) heterogeneities in the network topologies are indicated by the demands of air travel for the geographic areas in which the airports serves. Firstly, large air travel companies in mature air travel markets adapt a hub-and-spoke model to achieve a balance of travel time for customers and increase efficiencies in the use of transportation infrastructure. In this model, a single airport is assigned to a single hub or multiple hubs to form a regional inter-connected community [26]–[28], where “stop over” and “feeder” routes exist; connecting the small airports with low degree connections to a larger degree hub [29]. The locations of airport hubs are selected as the optimum locations that satisfy the inter-regional travel demands and minimize the total transportation cost [26], [27]. Moreover, the hub-and-spoke layout can be reflected on the “small-world” and the “scale-free” characteristics on the network. Guimera et al [30] studied the “small-world” feature and showed that most airports can be reached from every other with only a small number of connections. They also identified how central nodes with low degree connectivity play an important role for inter-regional and intra-regional communication. The “scale-free” feature ensures that the degrees of the air travel network follow a power-law distribution as suggested by the nodal structure of flows clusters [31] and described by the hierarchical span of the major airports in the United States [16].

Secondly, the connectivity and centrality of airports in the air travel network can act as indicators for air travel demand, since the local measurement of air passenger volume, population, and the level of economic activities at the periphery of the hub are highly correlated [32]–[34]. Empirical research [35]–[37] suggests a link between observed incremental growth of air passengers, increased passenger flows, and economic growth. Liu et al. [38] quantified the marginal effects of population growth in metropolitan areas on the air travel market, indicating that the odds of having a ‘major’ air traffic market increase 41% per 100,000 population growth. Wang et al [39] studied the air travel network in China and found that cities in the more urbanized area of East China had a higher centrality score and a higher number of air passenger volumes compared to the more rural West China. These studies indicate the mutual correlation of network centralities and urban development, and reflect the spatial agglomeration of economic activities and unequal air travel service demands.

To study the movement of vector-borne disease on the air travel network, Johansson et al [17], [40] modeled the actual passengers counts between 141 airports worldwide, for origins and destinations that had epidemic significance. Utilizing the air travel itineraries of the United States as a training set, they constructed a generalized linear model with a Poisson link to estimate worldwide passenger flows using nodes and routes characteristics as model covariates. Their models provided reasonable flow predictions of origin-destination travel. Our research follows the general modeling framework used in Johansson et al [17], [40], but extends the specification to a global model which includes: 1) all nodes with a host-city population of more than 100,000; 2) routes between all airports that are within 0, 1 or 2 stops on the air travel network.

## Materials and Methods

### Airport Locations and Scheduled Routes

Information on a total of 3,416 airports across the world, together with their coordinate locations was obtained using Flightstats (www.flightstats.com) for 2010. The connectivity and scheduled air travel network routes were defined by a 2010 scheduled flight capacity dataset purchased from OAG (www.oag.com). These included information on direct links (if a commercial flight is scheduled) of origin and destination airports, flight distances, and passenger capacity by month for 2010. Directly connected airports pairs were utilized to construct a graph for the air travel network in 2010 with 3,416 nodes and 37,674 edges. The average degree of the network was 22.06, with the maximal degree recorded as 476 for Frankfurt Airport (IATA code: FRA). The topology of the graph exhibited both small-world and scale-free properties as already observed in similar global or regional air travel dataset analyses [30], [41], [42]. The coefficients of the power law function fitting the scaled-degree distribution was 1.01±0.1, which is in concordance with a previous study [30]. The average path length is 4.11, measured as the average number of steps travelling from any one node to any other node, while the diameter of this network was 14 (which indicates the shortest path between the two most remote airports). Based on the network created by the flight statistics assembled, we calculated the degree, centrality and strength for each node and use these measurements as covariates at the modeling stage.

### GDP and Population Information

Generally, socio-economic variables at a global scale are difficult to obtain. The G-Econ data (http://gecon.yale.edu/) provide indices representing both market exchange rates (MER) and purchasing power parity (PPP) at a 1-degree longitude by 1-degree latitude resolution at a global scale. Due to the large geographical coverage of the grid cells, we extracted the closest PPP value for an airport and calculated the PPP value per capita in 2005 by dividing the purchasing power parity by the population value in each grid cell. These data were utilized as local economic measurements for each airport.

Given computing power limitations on the modeling and matrix sizes, we selected the airports serving a city population number more than 100,000. To select these airports, a web crawler built on the WolframAlpha API (http://products.wolframalpha.com/api/) was used to extract the city populations for each airport. Wolfram alpha is a knowledge engine which is capable of computing population information from various sources including: U.S census data, United Nations urban agglomeration and City Population (http://www.citypopulation.de/) data. These data capture the most recent city population estimates from these data sources (for cities in United States, the US Census 2010 data were utilized). In our database, there were 1,491 airports satisfying these criteria.

### Actual Travel Passenger Flow

Data on passenger origins and destinations on the air travel network were obtained from a variety of sources to construct a training dataset:

1. The DB1B market data from the Airline Origin and Destination Survey (DB1B) provides a 10% sample of U.S. domestic passenger tickets from reporting carriers, including information such as the reporting carrier, origin and destination airports, prorated market fares, number of market coupons, market miles flown, and carrier change indicators. To create a training dataset, these data were aggregated annually by the origin and the destination airport code with the sum of counts of itineraries. This sum of counts was simply multiplied by 10 to reflect the 10% sample schema. To protect the US air travel industry, the reported international Origin-Destination data by the U.S carriers is strictly restricted to U.S citizens, and requires detailed statements on the use of the data. Hence, the research presented here did not take into account the international portion of the Origin-Destination data from DB1B.
2. The Canadian transport department provides statistics relating to the movement of aircraft, passengers and cargo by air for both Canadian and foreign air carriers operating in Canada (http://www23.statcan.gc.ca/imdb/p2SV.pl?Function=getSurvey&SDDS=2703&lang=en&db=imdb&adm=8&dis=2). This survey provides estimates of the number of passengers traveling on scheduled domestic commercial flights by directional origin and destination city pairs. In this survey, significant numbers of Canada-U.S trips were reported. The city pairs were matched to the airport pair that had the shortest routes defined by the OAG database with the passenger number obtained from the above data source. For example, passenger numbers between Toronto and New York City were matched to the direct route of YYZ to JFK, since it is the shortest route between these two cities.
3. Detailed route data for passenger numbers from EuroStat (http://epp.eurostat.ec.europa.eu/portal/page/portal/transport/data/database ). This database presents passenger numbers between the main airports of reporting countries and their main partner airports in the European Union.

All of these flow statistics were utilized to create a training dataset O-D matrix. In this training dataset, there were 95,709 aggregated itineraries between 712 airports. The covariates used for modeling are described below.

### Network Covariate Processing

Cities are situated in a complex hierarchical network and the flows between cities are either constrained or facilitated by this hierarchical structure [4], [43]. We defined three levels of economic activity for each city per capita based on the 33% quartile of the distribution of PPP per capita. Thus, nine types of economic links were identified (low-low, low-medium, low-high, etc.) to reflect the type of flow within/across the economic hierarchies. Similarly, we defined four levels of hierarchy based on the degree distribution of the airports, and sixteen types of flows were identified to reflect the type of flow within/across the air service hierarchies.

A prediction dataset framework for routes was constructed based on the adjacency matrix defined by the OAG dataset. For each airport, destination airports via first-order connection, second-order connection and third-order connections on the air travel network were identified. Along these routes, information on the minimum number of stopovers and the maximum seat capacity were calculated. Moreover, following approaches outlined in Bhadra’s research [32] we defined a categorical variable for distance classes to separate the markets by stage lengths, with 1 for short-haul (2,000 kilometers or less), 2 for medium-haul (between 2,000 and 3,500 kilometers) and 3 for longer hauls (3,500 or more kilometers). We excluded routes less than 200 km since passengers are believed to have more efficient and effective land-based methods to travel such small distances. Note that only 3,842 possible routes (<0.001%) are less than 200 km. Finally, an origin-destination (OD) pair list with 1,295,752 rows was created.

For analytical purposes, the global OD pair list was constructed following these assumptions:

1. Passengers always take the shortest path to their destination city, and they don’t stop at the connecting city. The data used for modeling is itinerary data which represents the minimum number of stops from one airport to another. Hence, passenger numbers in our database represented the flows for the first order, the second order and the third order of network connections. We assumed that passengers choose the first shortest path found by a breadth-first search algorithm, as the route was found by iterating all the neighboring nodes until a path from the origin and the destination was identified. If both the origin and the destination cities have multiple airports, the passengers were assumed to take the shortest path from all possible routes between these airport pairs, which usually resulted in the path between the two largest airports in terms of capacity. This assumption is supported by Button et al [44]’s research that passengers tend to choose a larger hub for their travel.
2. Passengers do not choose routes with more than two stops. We used the number of stops as a categorical variable rather than a numeric variable since it is considered to be a measure of hierarchical accessibility. In fact, for the air travel network in 2010, all of the possible calculated routes within two stops covered 83% of all the possible connections. Also, multiple-stops (more than two stops) were comparatively rare as a share of total passengers in our actual travel flow datasets. In DB1B domestic datasets, there are no itineraries for travels between cities with a population size more than 100,000 within two stops.

All the network characteristics were calculated using the igraph (http://igraph.sourceforge.net/) library in R (http://www.r-project.org/). Snowfall library (http://cran.r-project.org/web/packages/snowfall/index.html ) was utilized for parallel processing to accelerate calculations. A summary of variables included in the model is presented in Table 1.

**Table 1:** Descriptions of covariates used in the modeling process.

Variables	Descriptions
*Node characteristics*	
Pop_i_	The population of the origin city
Pop_j_	The population of the destination city
PPP2005_i_	The purchasing power index where the origin airport serves
PPP2005_j_	The purchasing power index where the destination airport serves
PDA2005_i_	The purchasing power per capita index where the origin airport serves
PDA2005_j_	The purchasing power per capita index where the destination airport serves
Strength_i_	The sum of the edge weights of the adjacent edges for each vertex for the origin city
Strength_j_	The sum of the edge weights of the adjacent edges for each vertex for the destination city
Degree_Out_i_	The degree number of the origin city on the air travel network
Degree_In_j_	The degree number of the destination city on the air travel network
Closeness_Centrality_i_	The mean geodesic distance between a given node and all other nodes with paths from the given node to the other node. This variable is calculated according to the origin city.
Closeness_Centrality_j_	The closeness centrality measure for the destination city.
Betweeness_Centrality_i_	The number of shortest paths going through the original airport.
Betweeness_Centrality_j_	This is the calculation of betweeness centrality for the destination airport.
*Route characteristics*	
Inverse Distance	Inverse great circle distance between the origin and the destination airport
Country	Indicates whether the origin and the destination are in the same country.
Alternative	Number of alternative routes to the destination
Stops	Number of stops on the shortest route from the origin to the destination
MaxC	The maximum capacity along the shortest path
Degree Link Type	This variable identifies the types of flows between different hierarchies of airports defined by the air travel services level.
Economic Link Type	This variable identifies the types of flows between different hierarchies of airports
Haul Type	This variable differentiates the effect of long haul flights. 1 for short-haul (2000 kilometers or less), 2 for medium-haul (between 2000 and 3500 kilometers) and 3 for longer hauls (3500 or more kilometers).

### Model

We firstly constructed and tested a model based on our training dataset and then applied the model to predict OD pairs globally. The model we utilized takes the form of a spatial interaction model:
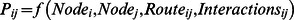
where *Pij* is the annual aggregated passenger flow between node i and j. *Node_i_* and *Node_j_* denote the collection of node characteristics, which are considered to be drivers of the size of the flow. *Route_ij_* denote the collection of route characteristics, which are considered to be the proximity measurements. *Interactions_ij_* denotes the collection of two-way interaction effects between categorical variables such as stops, country, degree link type, economic link type and haul type, as well as other node and route characteristics.

For the purpose of enhancing estimation and thus prediction, we tested four model specifications which include 1) a lognormal model for main effects only. This model adopts the general gravity model framework as the one described in Balcan et al [45]. To utilize this model, a logarithm transformation is performed for each quantitative variable. The main effects included both node and route characteristics. 2) A generalized linear model for main effects and interactions with Poisson distribution and a log link. This model adapted the model utilized by Johansson et al [17], [40] for predictions of the traffic flows between epidemiologically significant cities. 3) A generalized linear model for main effects and interactions with a negative binomial distribution and a log link. This model is similar to model 2 except that it utilized a negative binomial distribution to account for the possible over-dispersion in the data. 4) A lognormal mixed model with main effects, interactions, and random effects on origin and destination city (note that a logarithm transformation is performed for each quantitative variable as well). This model assumed that the passenger flows were independent between different degree link types but correlated within the same degree link type, while model 1–3 made the assumption that all passenger flows were independent of each other, which is very strong and unrealistic in practice. Random effects were thus included to account for the dependence among passenger flows and the possible heterogeneity between levels of air travel services. More detailed model descriptions can be found in Text S1.

Apart from model fitting on the entire training dataset, cross-validation was performed to evaluate how accurately each model would predict in practice: firstly, the training dataset was randomly partitioned into 10 subsets, each consisting of 10% of the observations. Then on each of the subsets (the cross-validation testing set), we validated the analysis using the remaining data. Lastly, the validation results were averaged over the rounds, with ranges of percentages reported. Three criteria were chosen for model evaluation: 1) the coverage rate of the 95% prediction intervals, which measured the percentage of the observations that fall into the corresponding 95% prediction intervals; 2) the coverage rate of the ±30% observation intervals, which measured the percentage of the predictions that fall into the ±30% intervals of the corresponding observations; 3) the successful prediction rate, which measured the percentage of predictions that fall into the same magnitude category as the corresponding observations. These magnitude categories were defined by dividing the passenger flow numbers into five groups: 10^2^ and under, 10^2^–10^3^, 10^3^–10^4^, 10^4^–10^5^, and 10^5^+, each group represents one category.

## Results

### Model Comparison on the Training Dataset

For each model, most coefficients were significant at the 0.05 significance level as the percentages of the significant coefficients are about 90%, 100%, 96%, 95% respectively for model 1 to 4. For the purpose of prediction, we kept all the covariates in the model instead of removing the non-significant ones. Not surprisingly, most of the interactions between node and route characteristics played an important role in model estimation as we treated the number of stops as a categorical variable. The interaction between haul types and inverse distance was also significant, which agreed with previous work [32].

Both model 1 and model 2 provided narrow confidence intervals for predictions, while model 3 and model 4 provided wider intervals to accommodate variation in the data. All of these models had at least 68% successful prediction rates for predicting the magnitude of passenger flow. According to the results presented in Table 2, model 4 provided the most accurate prediction.

**Table 2:** Comparison of the four models with respect to prediction accuracy (in percentages).

	Coverage rate of the 95% prediction intervals	Avg. coverage rate of the 95% prediction intervals(cross-validation)	Range of coverage rate of the 95% prediction intervals(cross-validation)	Coverage rate of the ±30% observation intervals	Avg. coverage rate of the ±30% observation intervals(cross-validation)	Range of coverage rate of the ±30% observation intervals(cross-validation)	Successful prediction rate	Avg successful prediction rate(cross-validation)	Range of successful prediction rate(cross-validation)
Model 1	6.39	6.73	[5.89,7.56]	29.82	29.76	[21.94,31.84]	68.42	68.33	[66.49,69.52]
Model 2	4.80	4.79	[0.27,10.33]	31.48	30.63	[29.62,31.82]	69.16	68.80	[67.44,70.34]
Model 3	23.16	24.16	[22.42,25.27]	33.09	33.38	[29.94,42.29]	70.04	69.43	[68.25,70.92]
Model 4	52.11	49.86	[46.97,51.63]	47.79	31.17	[30.52,33.2]	79.72	70.41	[69.97,72.71]

For each of the models, we calculated the Root Mean Square Error (RMSE) and Mean Absolute Error (MAE). RMSE is a frequently used measure of the differences between estimate values and the values actually observed. A smaller RMSE suggests a better model fit. MAE is the average of the absolute value of the prediction errors, which serves the same purpose as RMSE and is believed to be more robust in many situations. As shown in Table 3, model 4 yielded the lowest RMSE and MAE for the majority of the data points except for extremely large observations. For the largest observed passenger value category, model 2 gave the lowest RMSE and MAE, while model 4 gave the second lowest RMSE and MAE.

**Table 3:** Root Mean Squared Errors and Mean Absolute Errors for all models.

Measurement	Categories	Number of Records	Model 1	Model 2	Model 3	Model 4
RMSE	Observed Passenger (OP)<10^2^	2379	1680	2923	1947	726
	OP in 10^2^–10^3^	6440	3536	5127	32405	1802
	OP in 10^3^–10^4^	7314	7397	8771	10639	4346
	OP in 10^4^–10^5^	4817	20780	23002	41585	21940
	OP>10^5^	1132	163352	85897	216610	127194
MAE	Observed Passenger (OP)<10^2^	2379	286	538	402	120
	OP in 10^2^–10^3^	6440	629	1073	1413	333
	OP in 10^3^–10^4^	7314	2729	3218	3140	1621
	OP in 10^4^–10^5^	4817	14697	14929	19689	13415
	OP>10^5^	1132	115710	61305	94447	89233

**Figure 1:**
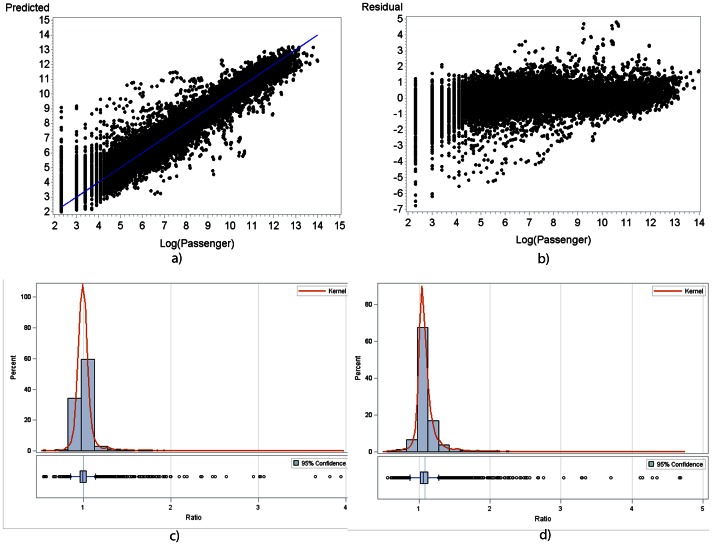
Diagnostic plots for all models. a) Predicted vs. observed value of model 4. b) Residual vs. observed value of model 4. c) Distribution of ratio of predicted value vs. observed value in log scale with 95% confidence interval for geometric mean. d) Distribution of ratio of capacity vs. observed value in log scale with 95% confidence interval for geometric mean.

Figure 1 presented the prediction and diagnostic plots for Model 4. In figure 1, panel a) showed that most of the prediction values are close to the y = x (prediction = observation) line; panel b) showed that most of the residuals scatter along the y = 0 (residual = 0) line, yielding no obvious pattern. Both plots indicated that Model 4 was a plausible model for the passenger flows. However, the prediction seemed poor at the lower tail. This was expected, given likely randomness in the smaller amount of passenger exchanges between airports [17]. Diagnostic plots for other models are presented in figure S1 and S2.

Alternative diagnostics for testing the model fit were performed for model 4 as well. Firstly, a multilevel model described in Snijders et al [46] and implemented in the SAS code written by Recchia et al [47] to calculate r-squared measures for the fourth model was utilized. The first level of the model which considered only the individual connectivity was found to explain 84.0% of the variance in the data, and the second level, which incorporated the independency between different degree link type group and the within-group correlation explained 98.7% of the variance, indicating a good model fit, and an improved explanation power in terms of variance. Secondly, for the directly connected flights, we compared both the predicted value from model 4 and the capacity data from OAG to the observed passenger flows on a log scale using paired t-test. The results showed evidence of difference between the mean predicted passenger number and mean observed passenger number, and between the mean capacity number and mean observed passenger number, both at the 0.05 significance level. However, the geometric mean ratio of log (predicted value) to log (passenger number) was 1.01(panel c) in Figure 1), while the geometric mean ratio of log (capacity) to log (passenger number) was 1.08(panel d) in Figure 1). The predicted values showed more agreement on the observed value, while the capacity data represented a significant overestimation of flows between two directly connected airports. Hence, our predicted values provided a closer approximation of the traffic flows on the air travel network compared to the maximum seat capacity metric for the directly connected cities, as used in previous studies [9]–[11], [13]–[16].

In summary, our model (Model 4) outperformed the lognormal spatial interaction model (Model 1) used in Balcan et al [45] and the Poisson model (Model 2) used in Johansson et al [17], [40] for the training dataset. Moreover, for direct flights, our estimates showed more homogenous agreements with observed passenger numbers compared to simple seat capacity data.

### Prediction and Interpretation of the O-D Passenger Flow Matrix

Model 4 was applied on the estimation dataset to predict passenger flows with coefficients extracted from the training datasets. We have identified the over-dispersed predictions that exceeded the maximum capacity on the routes (3% of the data) and replaced them with the product of the maximum capacity on the routes. According to the training dataset, the maximum numbers of itineraries for one-stop and two-stop connections were 140,086 and 8,060, respectively. Since these data were generated from the mature air travel market and constrained by the network structure, we considered them as the upper limits of the data distribution. As such, we adjusted the prediction of the first-order connection and the second-order connection flights scaled by these two maximum numbers. We then removed all predictions that were less than 1 person. Finally, 644,406 routes with origin/destination airport codes, number of stops, and predicted passenger numbers were produced.

**Figure 2:**
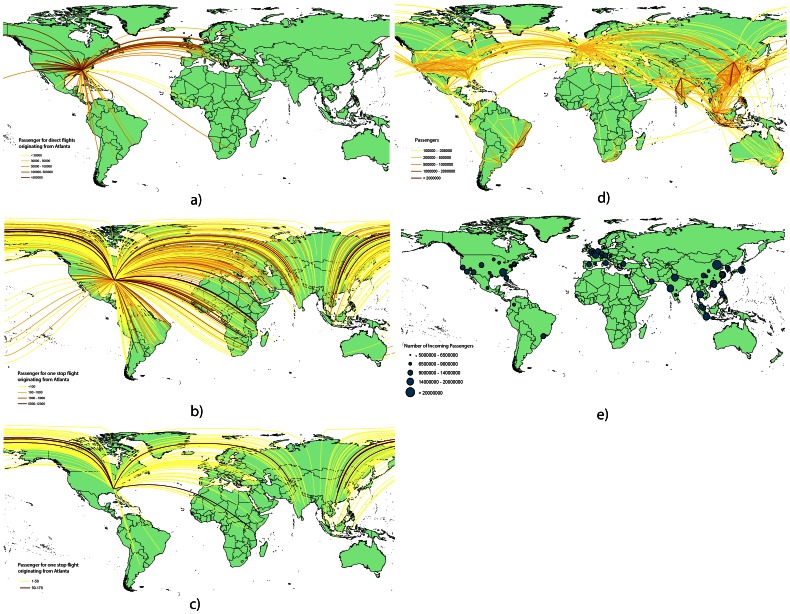
Predicted air traffic flows. a) Predicted flights with passenger flows of more than 100,000. b) All possible passenger flows through direct flights originating from Atlanta. c) All possible passengers’ flows through one-stop flights originating in Atlanta. d) All possible passengers’ flows through two-stop flights originating Atlanta. e) All airports with an incoming passenger numbers more than 5,000,000.

As described before, the passenger counts were grouped into five categories as a test of successful prediction rate in magnitude: 1–10^2^,10^2^–10^3^,10^3^–10^4^,10^4^–10^5^and 10^5^ and more. The first two categories presented small numbers of passenger exchanges, implying random flows between two airports, and the fourth and fifth categories indicated a higher probability representing steady flows between airports. Figure 2 a) showed all the flows with more than 10^5^ predicted passengers.

Secondly, given an origin/destination, the dataset produced through the research outlined here can estimates the endpoints and starting points with passenger flows on the air travel network. Figures 2 b)-d) illustrated the passenger flows and number originating from Atlanta, categorized by number of transfer. Figure 2 e) showed the distribution of airports with incoming passenger numbers over 5,000,000. This reflected the mature air markets of the United States and Europe, though noticeable concentrations of airports could be observed in the emerging markets such as India and China as well.

## Discussion

With continuing growth of the global air travel network, we must expect continued socioeconomic, environmental, cultural and epidemiological impacts. This research shows how network characteristics combined with multiple datasets on various perspectives relating to the movements of passengers of passenger flow on the global air network can be compiled to provide estimates that are more accurate than previous modeling efforts. Such a dataset provides a valuable resource for scientists and decision makers to measure the global flow of air traffic and its potential influences.

In the database outlined here, 644,406 unique routes spanning 1,491 airports serving city populations of more than 100,000 were modeled based primarily on publicly available datasets. On the training dataset, our model has outperformed similar research at the global scale and can explain 98% of the variance in the data. Within the database, 23,785 routes follow a direct connection, 291,745 routes are one-stop connections and 328,876 routes are two-stop connections. Using this route and airport information, anyone can construct flow matrices to describe the global air traffic flow and assess its multiple impacts.

Due to data constraints, a range of uncertainties and limitations exist in the output modeled datasets. The first inconsistence comes with internal uncertainties within the DB1B dataset. To construct the DB1B dataset, Transtat only requires US carriers to report O-D pair data, hence the O-D data is likely to be inaccurate in markets served with a significant number of foreign carriers (e.g., New York, Washington D.C., Chicago, and Los Angeles).Meanwhile, flights operated by foreign carriers usually have a share code with U.S. carriers and these flights are included in our database. If there is more than one airport in a city, each of the airports is treated as a separate node. This may well result in overestimates of the flow to secondary airports in a city.

The second set of inconsistency is the population data. Due to data availability, only city population data were utilized, when it is sometimes the case that people in neighboring metropolitan area can access the airport in question through other ground-based transportation methods (for example, people in Gainesville FL are often likely to drive two hours to Jacksonville or Orlando to take a plane, rather than utilize the Gainesville regional airport which is 10 miles away from the city center). As a result, our predictions may overstate the markets for small airports.

The third source of uncertainty stems from the fact that the data we utilized for the training datasets were only from the United States, Canada and the European Union. Thus, international flights are less well represented in our dataset and most of the flight data describes the flows between airports in high income countries. Additionally, long haul international flights with more than three stops are absent.

The topology of air travel network is likely to vary at the regional level. Wang et al [39] found that in terms of topological measurements, the Chinese air travel network is similar to the Indian one, but different than that of the US. As current air travel networks in low income countries usually feature point-to-point connections between city pairs [48], high income countries are increasingly prompted to utilized a hub-and-spoke system due to their mature air travel markets. On the other hand, it is observed that some companies (such as Southwest Airlines and Jet Blue in United States) in high income countries also adopt spoke-to-spoke models to connect hot spots of air travel demand [32]. This heterogeneity may affect the flow estimation country-wise and overestimate the driving factor of hubs in both high and low income countries.

The demand for air travel are heterogeneous and “largely determined by the spending capacity of customers” [49]. Hence, it could be anticipated that the demand for air travel in each country varies and is correlated to GDP. Also, the demographic profile of passengers on the air travel network is likely different between countries. Under a regional context, this may affect the prediction of domestic passenger numbers, while international heterogeneities in traffic flows may be attributed to differing visa policies between countries [50]. Visa restrictions may reduce traffic flows substantially between countries [51]. Moreover, cultural differences at a country level could represent indicators of attraction and drivers of population movements [6], [52].

The potential limitations discussed above arise through the constraints of the data sources used. These may be alleviated through incorporation of more publicly accessible data in future work, including: 1) more detailed economic indicators (such as GDP, income etc.) at the city level: such measures could further describe drivers in the spatial interaction model; 2) itineraries from low income regions of the world–such data would enlarge our training and testing databases to avoid sampling errors; 3) hub characteristics (such as the number of enplanements, transfers and deplanements): these measures could help explain the function of the hubs in controlling network flows. Alternatively, transportation forecasting models [53], [54] and mobility and migration models [55] could be utilized to estimate the global O-D matrix based on the traffic counts on nodes and edges.

### Conclusion

The research presented here has documented the generation of a world-wide Origin-Destination matrix of passenger flows in 2010 for airports with host city populations of more than 100,000. Results show that the modeled dataset improves substantially on the accuracy of datasets used in previous studies. The datasets are freely accessible for academic use and are published as part of the Vector-Borne Disease Airline Importation Risk (VBD-Air) project at www.vbd-air.com/data/.

## Supporting Information

Figure S1 **Plots for predicted value vs. the predicted value at a log scale.**
(PNG)

Figure S2 **Plots for residuals vs. the predicted values at a log scale.**
(PNG)

Text S1 **Model description.**
(DOC)
