# Supplementary figures and images for: An Open-Access Modeled Passenger Flow Matrix for the Global Air Network in 2010
Source: PLoS One. 2013 May 15;8(5):e64317. doi: 10.1371/journal.pone.0064317 (PMC3655160; doi:10.1371/journal.pone.0064317)

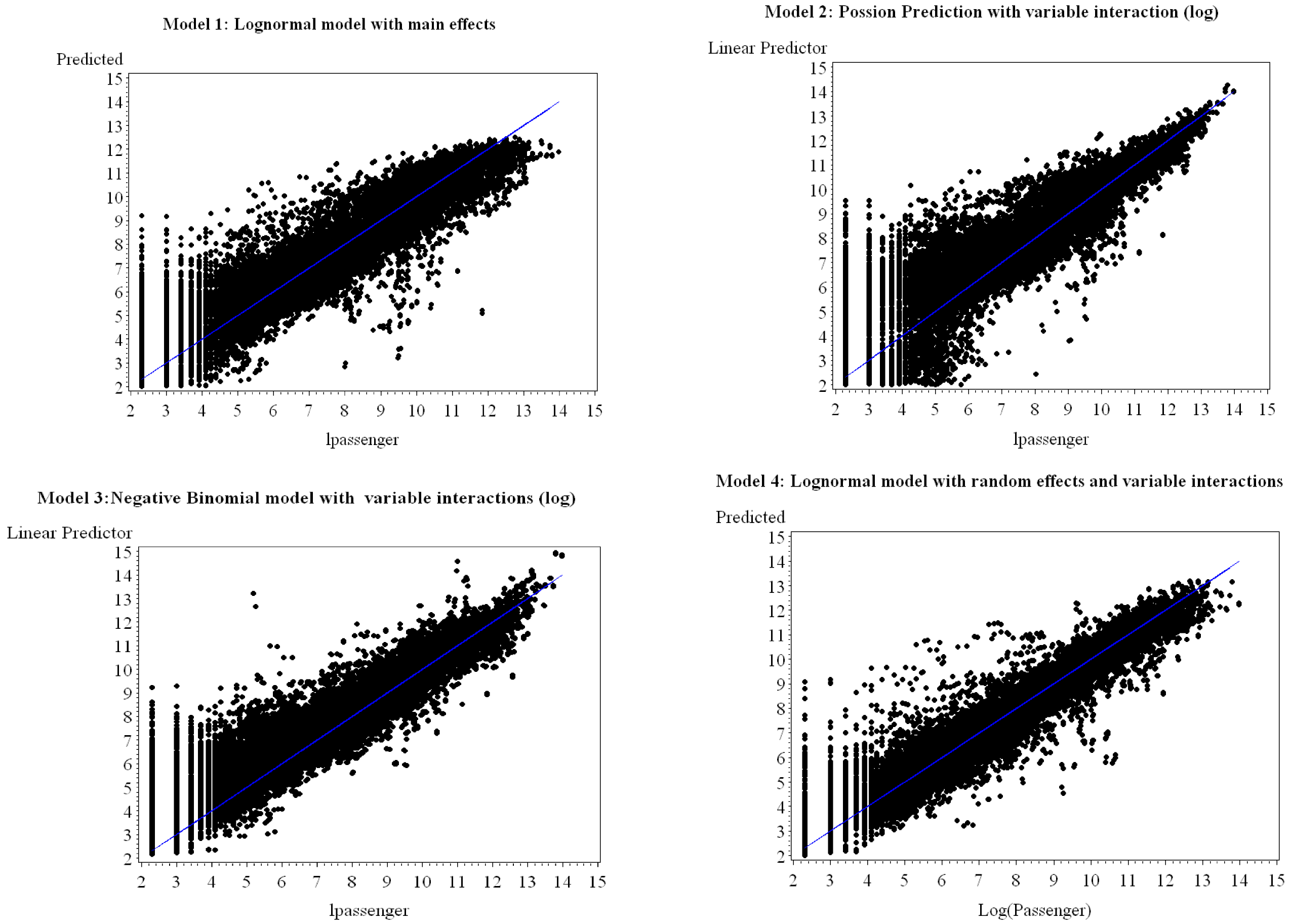

Supplement: Figure S1 — Plots for predicted value vs. the predicted value at a log scale. (PNG) [file pone.0064317.s001.png]

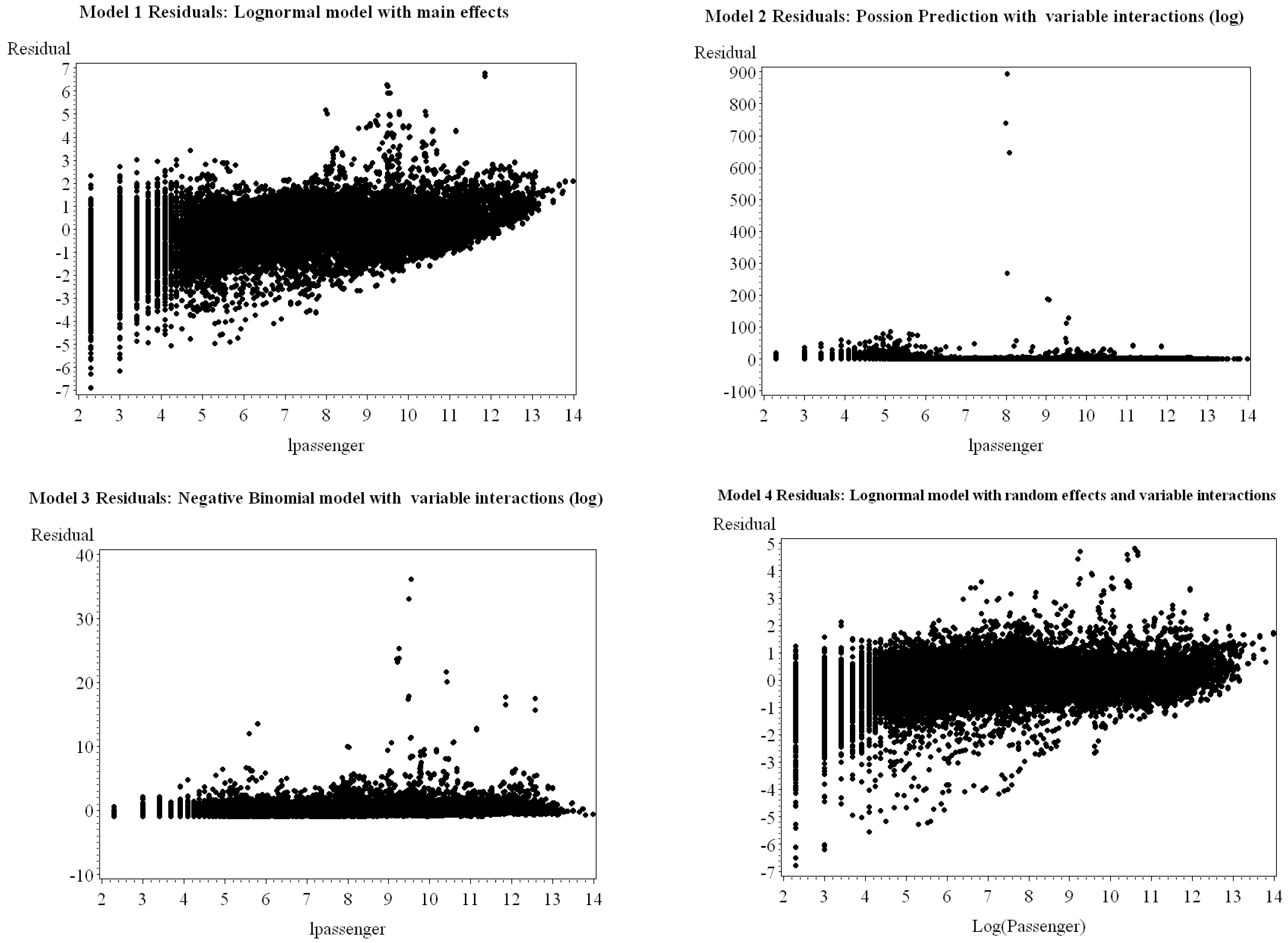

Supplement: Figure S2 — Plots for residuals vs. the predicted values at a log scale. (PNG) [file pone.0064317.s002.png]
