# Supplementary material for: An Open-Access Modeled Passenger Flow Matrix for the Global Air Network in 2010
Source: PLoS One. 2013 May 15;8(5):e64317. doi: 10.1371/journal.pone.0064317 (PMC3655160; doi:10.1371/journal.pone.0064317)
Supplement: Text S1 — Model description. (DOC) [file pone.0064317.s003.doc]

Model Description

To choose the best model for the predictions of air travel number, we adopted and compared these four models:

1. Lognormal model


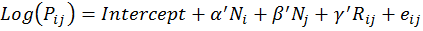


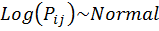


1. Poisson model with variable interactions


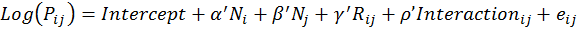


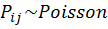


1. Negative Binomial Model with variable interactions


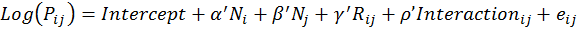


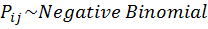


1. Loglinear model with variable effects and random effects on origin and destination airports


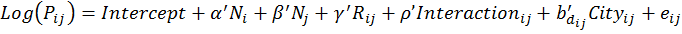


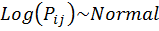


In these models,
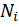
 and
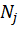
 denote the vector of node characteristics for the origin airport i and the destination airport j.
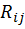
 denotes the vector of route characteristics.
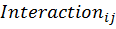
 denotes the vector of the two-way interactions between categorical variables such as stops, country, degree link type, economic link type and haul type with other node and route characteristics.
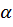
,
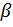
,
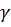
 and
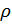
 are model coefficients. The first model utilizes a lognormal model with all the variables transformed on a logarithm scale. The second model utilizes a generalized linear model framework with a log link and Poisson distribution. The third model utilizes a generalized linear model with a log link and Negative Binomial distribution. The fourth model utilizes a lognormal mixed model with main effects, interactions, and random effects on origin and destination city. In this model,
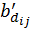
 denotes the vector of random effects on origin and destination city.
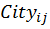
 denotes the vector of origin city i and destination city j. Figure S1 shows the prediction plots for all four models. Figure S2 shows the residual plots for all four models.
